# Impact of Nesting Mortality on Avian Breeding Phenology: A Case Study on the Red-Backed Shrike (*Lanius collurio*)

**DOI:** 10.1371/journal.pone.0043944

**Published:** 2012-08-28

**Authors:** Jan Hušek, Karel Weidinger, Peter Adamík, Tore Slagsvold

**Affiliations:** 1 Centre of Ecological and Evolutionary Synthesis (CEES), Department of Biology, University of Oslo, Oslo, Norway; 2 Department of Zoology and Laboratory of Ornithology, Palacký University, Olomouc, Czech Republic; 3 Museum of Natural History, Olomouc, Czech Republic; Hungarian Natural History Museum and Eotvos University, Hungary

## Abstract

The seasonal timing of avian reproduction is supposed primarily to coincide with favourable feeding conditions. Long-term changes in avian breeding phenology are thus mostly scrutinized in relation to climatic factors and matching of the food supplies, while the role of nesting mortality is largely unexplored. Here we show that higher seasonal mean daily mortality rate leads to a shift in the distribution of breeding times of the successful nests to later dates in an an open-nesting passerine bird, the red-backed shrike *Lanius collurio.* The effect appeared to be strong enough to enhance or counteract the influence of climatic factors and breeding density on the inter-annual variation in mean hatching dates. Moreover, the seasonal distribution of reproductive output was shifted to larger, or smaller, broods early in the season when the nesting mortality increased, or decreased, respectively, during the season. We suggest that population level changes in timing of breeding caused by a general advancement of spring and of the food supplies might be altered by the seasonality in nesting mortality. Hence, we argue that consideration of nesting mortality is of major importance for understanding long-term trends in avian phenology, particularly in species capable of renesting.

## Introduction

Optimal timing of reproduction is important from both the life-time and the seasonal perspective. In seasonal environments, animals may often benefit from an early time of breeding [1], [2], [3]. Experimental studies suggest that seasonal date causally affects fitness of reproducing animals [4], [5], [6], [7]. In a wider geographical comparison, a main proximate driver is the seasonal prolongation of the photoperiod affecting timing of reproduction on the annual basis, especially at higher latitudes [8], [9]. On a more local basis, and at a finer scale, other seasonal factors are involved, in particular climate [10], [11]. Ultimately, reproductive success is maximized when the emergence of progeny coincides with periods of favourable food abundances [12], [13], [14], low population density [15], [16], [17], and favourable survival prospects for the progeny [18], [19], [20], [21] and the parents [22]. Among these, food abundance has been recognized to play a primary role, while other factors have received less attention, though they may also affect breeding recruitment of progeny to the subsequent population [1], [5], [17], [23].

Recently, many organisms, and particularly birds, seem to start reproduction earlier in the season as a result of higher spring temperatures [24]. However, some bird species do not seem to advance breeding fast enough to synchronize with the advancement of the peak in prey availability [25], [26]. Different hypotheses have been proposed for this mis-match, e.g. genetic constraints on commencing earlier breeding [27], a difference between the cues used by birds for the onset of breeding and the cues used by their prey for growth [28], and opposing selection on breeding dates from matching lower and higher trophic levels [25]. Indeed, the importance of any ultimate factor on the timing of breeding depends on the degree of its seasonality (i.e. seasonal variability). In environments where seasonality in resource abundance is less pronounced, other factors might become of major importance, such as conspecifics density and nesting mortality.

Lack [12] suggested a rather minor role of juvenile mortality for the timing of breeding in birds but some contradictory evidence comes from the cavity nesting great tits *Parus major* and coal tits *P. ater*. Seasonal increase in post-fledging predation by European sparrowhawks *Accipiter nisus* [29], [30] seems to generate directional selection on early breeding in these birds, the selection being particularly strong soon after the nestlings have left the nest [31]. Yet, open nesting species suffer from high predation pressure already during the nesting period [32]. Mixed evidence exists for the effect of nest predation on seasonal timing of reproduction, with some studies supporting Lack’s view [33], [34], [35], [36] while others do not [37], [38], [39], [40], [41], [42]. Morton [40] suggested that higher nest predation later in the season is the main factor causing tropical clay colored robins *Turdus grayi* to breed early, while Pienkowski [41] showed that ringed plovers *Charadrius hiaticula* may start egg laying relatively late apparently to avoid high nest predation early in the season. Seasonal increase, or decrease, in nesting mortality may thus select for earlier, or later, breeding, respectively. More recently, some studies have provided evidence that birds may delay egg-laying when the perceived predation risk is high [39], [42], although others have shown an opposite pattern even in the same species [38]. After a breeding failure, birds are also faced with the decision of whether or not to produce a replacement clutch [43]. This is because renesting often occurs after a nest failure, particularly early in the season, in order to secure reproductive output. All else being equal, the higher the general level of nesting mortality, the more breeding pairs will establish a replacement clutch. As a result, the overall seasonal distribution of breeding dates of a population will be shifted towards later dates.

In many species, inter-annual trends exist in mean seasonal nesting mortality [44], [45]. If breeding failures are common, and many individuals are able to renest quickly, this will strongly influence the apparent breeding phenology of the whole population. Hence, insights in nesting mortality/renesting dynamics are crucial to single out effects of other factors, such as global change of climate (increasing temperature, and phenological advancement of the food supply).

In Central Europe, an open-nesting passerine bird, the red-backed shrike *Lanius collurio*, L., recently tends to arrive earlier to the breeding grounds in Poland and Hungary [46], [47] and to start breeding earlier in the Czech Republic [48]. This seems to be related to increasing spring temperatures and/or breeding densities [16], [48]. However, in this species great variation and long-term changes have also occurred in nesting mortality [49]. Here we study long-term breeding data of this species, first (prediction #1) asking how the relationship between nesting mortality/renesting may have influenced the breeding phenology of the species over the years, controling for the effects of spring temperatures, and breeding density. Second (prediction #2), we test the hypothesis that a seasonal decrease in nesting mortality will shift the distribution of the reproductive output to larger broods occurring later in the season, whereas a seasonal increase in nesting mortality will give an opposite result. Hence, we study the interplay of three ultimate factors, namely seasonalities in general spring progress, nesting mortality, and breeding density, in determining the seasonality in the reproductive output of the red-backed shrike.

## Materials and Methods

### Red-backed Shrike Breeding Data

Breeding data were extracted from four sites across the Czech Republic (Table S1) from a combination of two datasets: (1) nestling ringing records stored at the Prague Bird Ringing Centre, and (2) field notes from bird ringers specialized at ringing shrikes [49]. We used data from those years for which we were able both to calculate estimates of nesting mortality (see below) and obtain hatching dates and brood sizes. Data were available for 15 years from site A (“Praha”) and B (“Masečín”), 42 years from site C (“Vítězná”), and 14 years from site D (“Vsetín”). At each site ringers searched for shrike nests regularly from middle May to early August, visiting the first active (i.e. after the first egg was laid) nest on average (±SD) on 29±11 May and making a final control of the last active nest on average on 23±11 July. The nests found were followed either until nest failure or ringing of the nestlings. Except one, each ringer was only active at one particular site per year. To avoid a possible bias introduced by unequal intra-annual searching effort, we excluded records from the sites from those years when the ringer was simultaneously working at more than one site. In order to obtain unbiased estimates of hatching dates of survived attempts we also only used data from years when the maximum length of the interval between successive nest visits at the respective study sites was less than 11 days (assuming a nestling period of 11 days as a minimum [50]). We ended up with data from 48 years, and with a mean annual number of nests with ringed nestlings of 36 (9–127) per site (in total 1728 nests, Table S2).

### Red-backed Shrike Population Density

Estimates of breeding density (number of breeding pairs per km^2^) for the respective study sites were available only for the period 1994–2006 from reports by red-backed shrike ringers and observers of the Czech Shrike Working Group [51]. The ln-transformed estimates were however strongly correlated with the respective ln-transformed numbers of ringed broods per km^2^ from our data (r = 0.81, p<0.001, n = 22). Therefore we used the ln-transformed number of ringed broods per km^2^ available for all years as a proxy for annual conspecific density (“ln(density)” hereafter).

### Spring Progress and Climatic Data

The rate of insect development and reproductive maturation during spring is critically determined by ambient temperatures [52]. Daily amount of accumulated heat when the temperature is above a given minimum threshold is often referred to as a “degree-day” (DD). Both insect and plant growth is strongly related to cumulated total of degree days during a period relevant for a given species [52]. We thus employed the cumulated total of DDs at a given date as a measure for general spring progress of the vegetation and of the shrikes’ insect prey [53].

Prey composition of adult and nestling red-backed shrikes is similar and consists mainly of insects, with Orthophera (up to ca. 44% of prey biomass), Coleoptera (up to ca. 23%), and Hymenoptera (2.5–4%) being the most important food items [50], [54]. Minimum temperature threshold for development of most insects lies within a range of 4–11°C, with minimal values recorded for Coleoptera (based on 55 species), Orthopthera (4 species) and Hymenoptera (91 species) of 3.7°C, 4.0°C and 2.4°C, respectively [52], [55]. Minimum temperature thresholds triggering bud growth, leaf unfolding and other tree phenophases lie somewhat lower than for insect development, within a range of 0–5°C [52], [56].

Following Schwartz [52], cumulated total of degree days at hatching day (CDD_HD_) for each nest in each year and at each site was calculated as a sum of the differences in mean daily temperatures and minimal developmental threshold temperature of 0°C from the start of the calculation period (1 January) to hatching date. We considered minimum threshold temperatures of 0, 2, 4, 10°C in analysed models but only present the best fitting model with threshold of 0°C. In central Europe, 1 January is relevant as a starting date for tree and even some Hymenoptera prey development and it is also used when there is no clear phenological event to start calculation from [52], [56], [57]. Mean daily temperatures at each study site were obtained from the respective closest meteorological stations (Praha-Libuš, ca. 7 km from site A; Neumětely, ca. 24 km from site B; Holovousy, ca. 19 km from site C; and Přerov, ca. 33 km from site D and Vsetín at site D). Mean May temperatures (T_MAY_ hereafter), i.e. temperatures during the time when the onset of breeding takes place, were also obtained. All climatic data were provided by the Czech Hydrometeorological Institute and the Research and Breeding Institute of Pomology Holovousy Ltd.

### Statistical Analysis

We were interested in general biological patterns. Therefore, we analyzed the relationships between explanatory and response variables across all sites, i.e. using all combined data and considering site as a random effect. We employed linear mixed models (LMM hereafter) and generalized additive mixed models (GAMMs). Generalized additive model (GAM) is a generalized linear model where the linear predictor of explanatory variables of the form ∑ β_j_(X_j_) is replaced by a sum of smooth functions with estimated degrees of freedom (“edf” hereafter) of explanatory variables ∑ s_j_(X_j_) [58]. Basis of the smooth functions is represented by thin plate regression splines (or similar) and is estimated as a part of fitting process. GAMM is an extension of GAM including random effects. We preferred to employ GAMM to LMM for two reasons in some cases: 1) it allows flexible modelling of nonlinear relationships between the explanatory and response variable by means of smooth functions, 2) in a graphical representation it directly shows the effect size of a given explanatory variable (i.e. partial residuals of a response) with its 95% confidence interval. We used nlme library [59] and mgcv library [58] in R 2.13.1 [60] to fit LMMs and GAMMs, respectively. We checked for normality of the response and explanatory variables and used ln-transformation when appropriate. Sample size was 48 site-specific annual values. We provide results of all respective analyses for particular sites employing linear regression in Results S1 and Table S3.

Using data from the Czech nest record scheme, Hušek and Adamík [61] showed that shrike nestlings are ringed on average when 7–8 days old, which is just a few days before earliest possible fledging age of 11 days, and that the ringed numbers closely match the numbers of fledglings from survived nests. Hence, we used all available ringing dates corrected for variation in age at ringing as a proxy for hatching dates of survived attempts. The age of 7 days at ringing was assumed if the exact age was unknown (25% of ringed broods). The number of ringed nestlings was used as a proxy for productivity (brood size).

Seasonal distribution of reproductive output was characterized by standardized differentials for hatching dates (SD_HD_). Standardized differentials were calculated by subtracting annual average hatching dates from the annual average hatching dates weighted for the brood size, and dividing this difference by the standard deviation of the hatching dates (see [62], [63]). Standardized differentials measure direction and strength of the skewness of the distribution of the reproductive output relative to the distribution of hatching dates. Negative/positive SD_HD_ indicates larger broods earlier/later in the season, respectively. The productivity of a particular nest presumably well reflects the annual productivity of a female and it does not seem to be seasonally confounded by female age or condition in this species [50], [64].

To calculate annual site-specific estimates of, and seasonal trends in, nesting mortality, we extracted data on nest inspections from the field notes of bird ringers at the respective sites (see “Nest monitoring data” in Table S2). We modelled nesting mortality in terms of daily mortality rate (“DMR” hereafter). A logistic-exposure method (an extension of logistic regression which allows for varying length of nest visit intervals [65]) implemented in the PROC GENMOD of SAS [66] was employed for estimation of DMR. The fate of the interval between successive nest visits is a binary response variable. Only nests with at least two visits were therefore considered (Table S2). Also, only nest visit intervals ending before the earliest possible fledging date were analyzed to avoid uncertain nest fates. For each year and site, we either modelled seasonally constant (mean) DMR or linear seasonal trends in DMR (“trendDMR” hereafter). Seasonal trend was modelled by including date (i.e. average date of each nest visit interval) as a covariate. To correct for annual variation, dates were expressed as deviations (i.e. relative dates) from the annual median clutch initiation date of all established nests, and standardized to zero mean and unit variance (for details on the data and analysis see [49]).

First, we analyzed inter-annual trends in variables. There was no need for formal time series modelling as there was no temporal autocorrelation in either first or mean hatching date (Durbin Watson test, all DW>1.7, all p>0.16), T_MAY_ (all DW>2.2, all p>0.16), trendDMR (all DW>1.8, all p>0.23) at either site A, C, or D (site B was not tested because of short time series). There only was indication of autocorrelation in the mean CDD_HD_ at site C (DW = 1.40, p = 0.027). Trends in DMR were analyzed elsewhere. Hušek et al. [49] found general increase in DMR from early 1980ties at three out of four sites.

Next, to test prediction #1, we fitted two LMMs on the relationship between mean hatching dates as response variable and T_MAY_ and DMR as explanatory variables. Then, in following two LMMs, we included year as a continuous explanatory variable to test whether the effects of T_MAY_ and DMR were confounded by the effect of inter-annual trend in mean hatching dates. Finally, we included T_MAY,_ DMR and ln(density) in a single complete LMM, and corroborated the results by fitting GAMM. There were no strong correlations between the explanatory variables (Results S2). The results and conclusions were similar for both first and mean hatching date and so we only present results for the latter one.

Last, to test prediction #2, we analyzed the relationship between the mean CDD_HD_, trendDMR and ln(density) as explanatory variables and standardized differentials for hatching dates (SD_HD_) as response variable in a single GAMM. The precision of estimates of DMR and trendDMR varied across years. When, however, the reciprocal variance of nesting mortality estimates was used as a weighting factor, the results did not change qualitatively and are therefore not presented.

## Results

### Inter-annual Variation

At each study site there was a large inter-annual variation in timing of breeding with a maximum range of 21 days for mean hatching date, and 22 days for first hatching date, respectively, particularly so at site C (Table S2). Estimated DMR also varied considerably among years, from 0.010 to 0.042 (Table S2).

**Figure 1:**
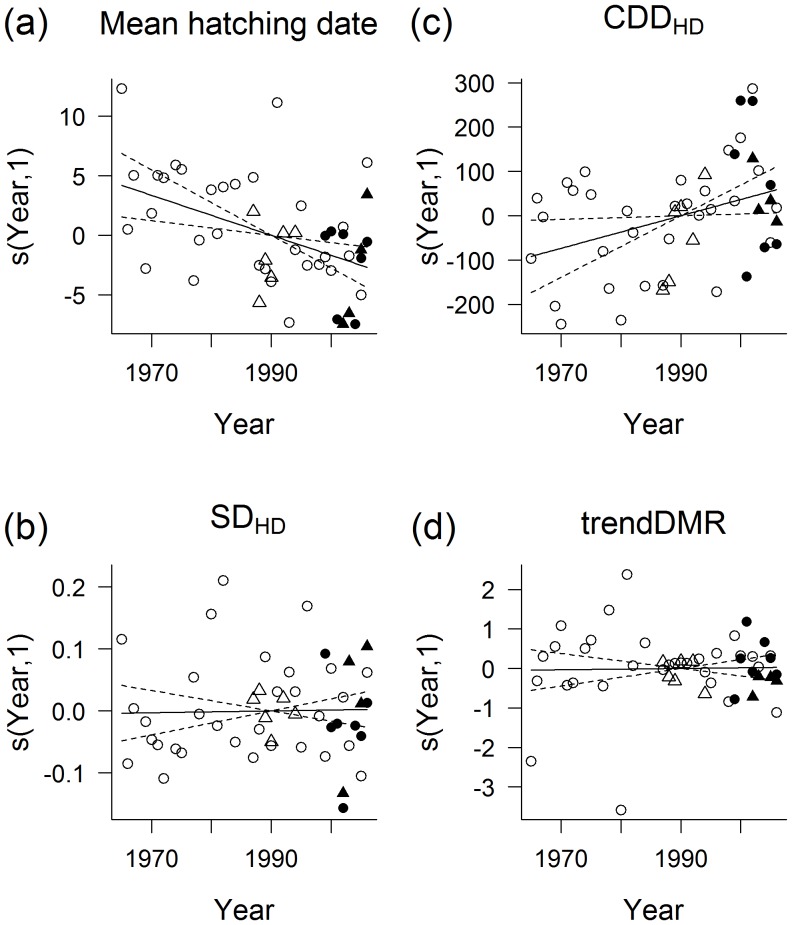
Temporal variability in selected breeding parameters of the red-backed shrike. Data across four sites in the Czech Republic during the period 1965–2006 were modelled by generalized additive mixed models (GAMMs) including year as a fixed continuous explanatory variable and the site as a random effect. The fitted smooth functions (“*s(covariate, edf)*” in the title of y axis) of the effect of year indicate its effect size with its 95% confidence intervals. Smoothing terms for the effect of year on a) mean hatching date: edf = 1, t = −3.12, p = 0.0033; b) standardized differential for hatching date (SD_HD_): edf = 1, t = 0.16, p = 0.87; c) mean cumulated total of degree days at hatching date with temperature threshold 0°C (mean CDD_HD_): edf = 1, t = 2.22, p = 0.032, similarly mean May temperature (T_MAY_) which is not shown; and d) standardized seasonal linear trend in daily mortality rate (trendDMR): edf = 1, t = 0.16, p = 0.87). Points are the partial residuals of the response (i.e. Pearson residuals added to the smooth term). The coincidence of the line of the estimated effect and its confidence intervals at the point where the line passes through zero for smooth terms with one degree of freedom is the result of the identifiability constraint applied for the smooth term (see [62] for details). Site A: full circles, site B: full triangles, site C: empty circles and site D: empty triangles. See Fig. S1 for site-specific trends and values on the original scales.

Across all sites, there was significant linear trend for earlier mean hatching date (LMMs with site as a random effect, fixed effects of year: b_year_ = −0.16±0.055, t = −2.95, p = 0.0052; Fig. 1a), and higher T_MAY_ (b_year_ = 0.06±0.020, t = 3.16, p = 0.0029) and mean CDD_HD_ (b_year_ = 3.66±1.653, t = 2.22, p = 0.0320; Fig. 1c) over the years. There tended to be an increase in ln-density during the years (LMM with site as a random effect: b_year_ = 0.01±0.005, t = 1.99, p = 0.05). There was no trend in either the standardized differentials for hatching dates (SD_HD_; b_year_ = 0.0001±0.0009, t = 0.16, p = 0.87; Fig. 1b), nor in the seasonality in nesting mortality (trendDMR; b_year_ = 0.002±0.010, t = 0.16, p = 0.87; Fig. 1d). See Fig. S1 for site-specific trends.

### Mean Hatching Date

Across all sites, the mean hatching date was significantly earlier when T_MAY_ increased (model a; Table 1). Conversely, mean hatching date delayed with higher DMR (model b; Table 1). When T_MAY_ was added as an additional covariate to a model with the effect of year, the trend towards earlier mean hatching dates was no longer significant across the sites (model c; Table 1). DMR, however, did not account well for the trend in mean hatching date in the model with the effect of year (model d; Table 1).

**Table 1:** Results of linear mixed models on mean hatching date in the red-backed shrike.

explanatory variable	slope	SE	t	p
*Linear mixed model a*				
**T_MAY_**	**−1.52**	**0.32**	**−4.71**	**<0.001**
Random effects variance: Intercept = 3.16, Residual = 3.57				
*Linear mixed model b*				
**DMR**	**185.52**	**73.53**	**2.52**	**0.015**
Random effects variance: Intercept = 2.76, Residual = 4.11				
*Linear mixed model c*				
**T_MAY_**	**−1.34**	**0.35**	**−3.79**	**<0.001**
Year	**−**0.07	0.05	−1.23	0.22
Random effects variance: Intercept = 2.90, Residual = 3.56				
*Linear mixed model d*				
**DMR**	**204.7**	**68.03**	**3.01**	**0.0044**
**Year**	**−0.17**	**0.05**	**−3.42**	**0.0014**
Random effects variance: Intercept = 1.69, Residual = 3.80				
*Linear mixed model e*				
**T_MAY_**	**−1.07**	**0.36**	**−2.98**	**0.0051**
**DMR**	**136.34**	**65.85**	**2.07**	**0.045**
Year	−0.09	0.05	−1.72	0.092
Random effects variance: Intercept = 2.59, Residual = 3.46				
*Linear mixed model f*				
**T_MAY_**	**−1.19**	**0.31**	**−3.90**	**<0.001**
DMR	91.21	62.19	1.47	0.15
**ln(density)**	**−3.46**	**1.03**	**−3.37**	**0.0016**
Random effects variance: Intercept = 1.49, Residual = 3.33				

**Figure 2:**
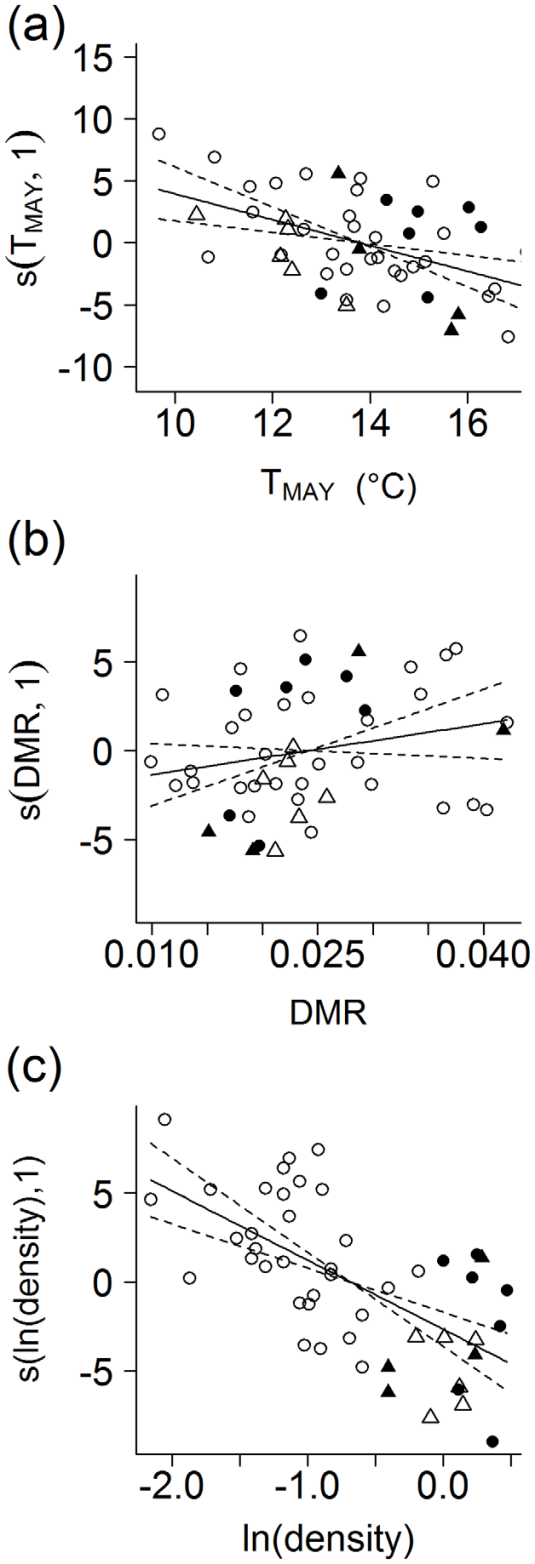
Effects of selected covariates on mean hatching date of the red-backed shrike. Modelled by GAMM was the effect of mean May temperature (T_MAY_), mean seasonal daily mortality rate (DMR) and ln-transformed breeding density [ln(density)] across four sites in the Czech Republic. Study site was included as a random effect. The fitted smooth functions indicate effect size with its 95% confidence intervals of a given factor (smoothing terms for the effect of T_MAY_: edf = 1, t = −3.50, p = 0.0011; DMR: edf = 1, t = 1.50, p = 0.14 and ln(density): edf = 1, t = −5.36, p<0.001). Partial residuals of the response are obtained by varying the effect of the explanatory variable concerned, while leaving all other variables fixed. See Fig. 1 for further explanations. See Fig. S2 for site-specific analyses and values on the original scales.

T_MAY_, DMR, ln(density) and year were included as fixed factors and site as a random effect (n = 48 annual values). Formal statistical significance at α = 0.05 is highlighted in bold. See legend to Fig. 2 for further explanations.

When both the effect of T_MAY_ and DMR were added to a model with the effect of year, mean hatching date still advanced (model e; Table 1). Mean hatching date still tended to be positively related to DMR after accounting for the negative effects of T_MAY_ and breeding density (see model f in Table 1; and Fig. 2). Predicted mean hatching dates for minimal and maximal DMR values of 0.010 and 0.042 (range in our study, Table S2) at mean values of remaining covariates were 172.00 (0 = 1 January) and 175.08, respectively. Observed range in DMR from 0.010 to 0.042 might therefore have resulted in a delay in mean hatching dates of about 3–4 days, respectively. See Fig. S2 for site-specific effects.

### Seasonality in Reproductive Output

**Figure 3:**
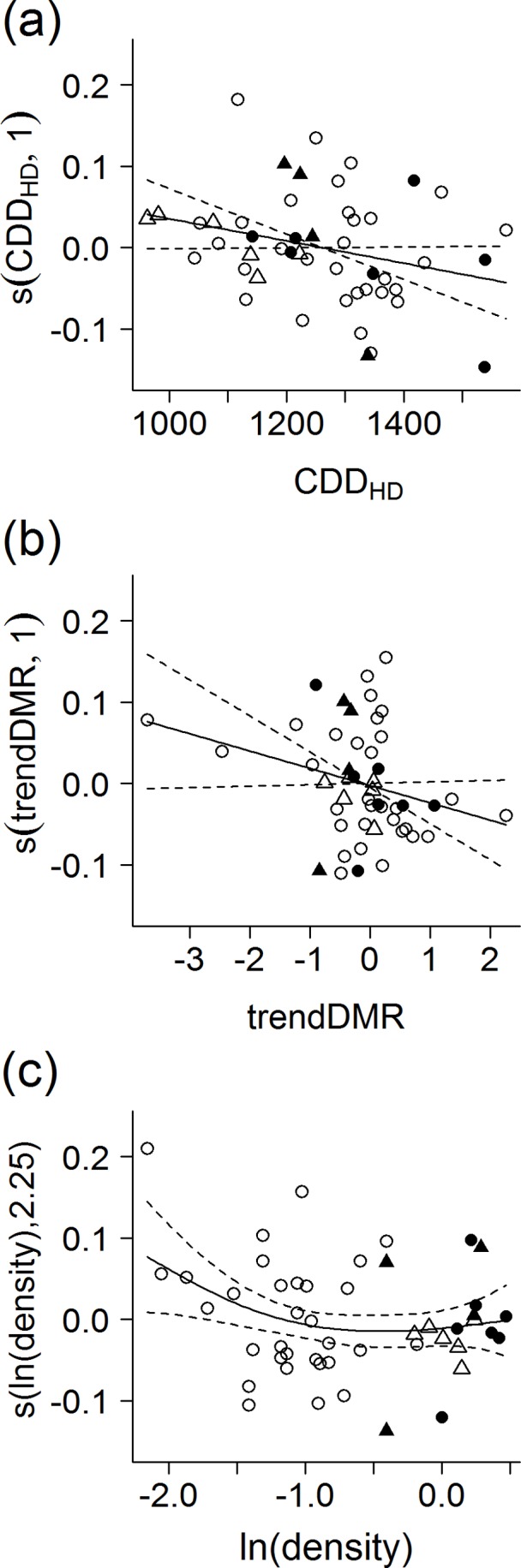
Effects of selected covariates on the seasonal distribution of reproductive output of the red-backed shrike. Modelled by GAMM was the effect of mean cumulated total of degree days at hatching date with temperature threshold 0°C (CDD_HD_), standardized seasonal linear trend in daily mortality rate (trendDMR), and ln-transformed breeding density [ln(density)] across four sites in the Czech Republic. Study site was included as a random effect. Seasonal distribution of reproductive output was expressed as standardized differentials between the mean hatching dates and mean hatching dates weighted by brood size. Smoothing terms for the effect of a) mean CDD_HD_: edf = 1, F = 3.70, p = 0.061; b) trendDMR: edf = 1, F = 3.40, p = 0.072; and c) ln(density): edf = 1.82, F = 3.22, p = 0.054. See legend of Fig. 1 and Fig. 2 for further explanations. See Fig. S3 for site-specific analyses and values on the original scales.

The distribution of reproductive output tended to be skewed to larger broods occurring earlier (negative values of SD_HD_)/later (positive values of SD_HD_) in the season when the spring progress was faster/slower at hatching times (Fig. 3a), when the the nesting mortality increased/decreased during the season (Fig. 3b) and when the breeding denstity was higher/lower (Fig. 3c), respectively. See Fig. S3 for site-specific effects.

## Discussion

### Significance of Nesting Mortality on Breeding Time

Here we showed that in red-backed shrikes, a species that readily establishes replacement clutches, higher seasonal mean nesting mortality would shift the overall distribution of hatching dates in a season towards later calendar dates. This effect was apparent also after accounting for the ambient temperatures prevailing during the onset of breeding, and for breeding density (Fig. 2). Hence, a general increase in nesting mortality after the early 1980ties (see Fig. 1 in [49]) may have been counteracting the effect of a general increase in spring temperatures, and a higher breeding density on annual variation in breeding times.

Admittingly, the demonstrated effect of the seasonal mean nesting mortality on the overall seasonal distribution of red-backed shrike breeding dates was rather weaker, i.e. with a shallower slope, than the effects of spring temperature and breeding density (Fig. 2). The annual variation in mean breeding time of the local populations amounted to three weeks (Table S2), whereas even high rates of nesting mortality only seemed to cause a delay of a few (up to 3–4, Fig. 2) days in annual mean hatching dates of all successful nests. Generally, the nesting mortality did not account well for the effect of year on mean hatching date while spring temperature did. Yet, the effect of nesting mortality appeared to be strong enough to enhance or even counteract the effects of other factors, particularly increasing spring temperatures, on the inter-annual variation in breeding dates. Breeding late within the season is generally assumed to be costly as it may reduce current and future survival prospects of both parents and offspring [7], [31], [67], e.g. because of temporal mismatch between breeding time and the time of peak food availability [2], [12], [13], [14]. Considering the effects of nesting mortality is therefore of particular importance in climate change studies in species that suffer high nesting mortality and that are readily capable of renesting. Many phenological studies focus exclusively on the distribution of first clutches. Our results show that the seasonal distribution of the total reproductive output may be affected by the proportion of replacement clutches.

The likelihood of renesting after having produced a successful brood is affected by food availability and the seasonal timing of nesting mortality [68], [69]. Similar factors probably affect renesting occurrence in many species where a replacement clutch is laid only if the first attempt fails [43]. On the contrary, exclusively single-brooded species, which are often long-lived, might postpone reproduction to the following year if the costs associated with renesting are too high [43], [70], [71].

Although, no inference can be reached on effect of nesting mortality on the timing of first clutches, it is clear that the seasonal shift in distribution of all breeding dates (first and replacement clutches pooled) also depends on the seasonality in nesting mortality. Similarly, based on life history modelling, Varpe et al. [3] have suggested that seasonality in predation (and resource abundance) affects reproductive prospects in a copepod *Calanoides acutus* by incurring seasonality in egg fitness. We built upon these finding by showing that distribution of reproductive output modelled as a difference between mean hatching date and mean hatching date weighted by the brood size shifted according to the seasonal pattern in daily mortality rate, spring progress and breeding density (Fig. 3). When the daily mortality rate was modelled as a linear function of the seasonal date, we showed that an increasing seasonal trend in nesting mortality was associated with larger broods occurring earlier in the season (negative standardized differentials for hatching dates), while a decreasing seasonal trend in nesting mortality was associated with larger broods occurring later in the season (positive standardized differentials, see Fig. 3b). In many years, there was no clear seasonal linear trend in daily mortality rate at all sites (see clump of data around value 0, Fig. 3b). Variability in estimates of seasonality in daily mortality rate in such cases increased considerably. As a result, some estimates appeared as outliers though those were biologically the most important ones. When only significant seasonal trends in daily mortality rate were analyzed, the pattern turned more apparent (Fig. S3b).

The effect caused by the seasonality in nesting mortality on the distribution of reproductive output (Fig. 3b) is comparable in strength to the one caused by the speed of the advancement of spring (Fig. 3a). The predicted standardized differentials for hatching dates for minimal and maximal values of the seasonality in nesting mortality at mean values of remaining covariates (−0.002 and −0.129) were similar to the predicted standardized differentials for minimal and maximal values of the spring progress (−0.037 and −0.121).

Non-mutually exclusive explanations exist to account for this observation because the distribution of the reproductive output on the population level may either mirror partial offspring mortality, seasonally plastic reproductive investment of individuals, or seasonal variability in timing of breeding of higher quality individuals rearing larger broods. The former might result from partial predation, selective predation on larger broods or partial mortality caused by deteriorated climate, food shortage or stronger competition. Whether or not birds adjust their seasonal reproductive effort in terms of timing of breeding, or clutch size, to seasonal patterns in nest predation, as determined by e.g. perceived predator density and activity, remains a major question [72], [73].

Recently, Decker et al. 2012 [74] did not find any support for the effect of seasonally decreasing nesting mortality rate on clutch size variation in red-faced warblers (*Cardellina rubrifrons*). In our study system, the association of larger broods occurring early in the season with seasonally increasing nesting mortality might simply be caused by decreased proportion of smaller replacement clutches in the population. This can not, however, explain the association of larger broods occurring later in the season with seasonally decreasing nesting mortality as larger replacement clutches are unlikely in red-backed shrikes [43]. Given a minor role of starvation and partial predation, our data rather suggest at least partial flexibility in brood size and/or seasonal variability in timing of breeding of higher quality birds according to seasonality in nesting mortality.

In the Czech subpopulation of the red-backed shrike, there is a trend over the years for spring to be more progressed at the time of hatching (Fig. 1c). Yet, there does not seem to be any comparable inter-annual trend in the seasonality of the nesting mortality (Fig. 1d), which might have possibly constrained any systematic shift in the annual distribution of the reproductive output (Fig. 1b). Inter-annually consistent patterns in seasonality in nesting mortality, e.g. caused by predator activity, seem to be rather rare and might often be counfounded by more random climatic effects. In general, juvenile mortality might be caused by abiotic [1] or biotic [5], [31] factors but predation is frequently identified to be one of the major factors causing nesting mortality in birds [32].

We did only consider mean annual estimates of breeding density in our study, though the fine tuning seasonality aspect might also be present, e.g. when caused by skewed distribution or arrival dates. Breeding density determines seasonal fitness primarily through competition for resources and altering of social interactions [17].

### Limitations of Studies on Nesting Mortality

In the studies of nesting mortality on timing of breeding, the main limititation is a demand for large annual sample sizes of nest failure data over many years because lower sample sizes cause less precise estimates of the seasonality trends. However, despite such limitations, we are confident in the main finding of our study, because the results obtained by including reciprocal variance of mortality estimates as weights in the model did not change qualitatively, though appeared a little weaker (or stronger) in some cases. Even if the effects were weak or formally not significant in the red-backed shrike population, they might be of major importance in other species suffering increased (or decreased) nesting mortality over the years in a rapidly changing world. Also, estimates of linear seasonal trend in DMR should rather be handled with caution because generally, data from the beginning of the season (egg laying, incubation) were scarce in our dataset (see [49]). We are however confident with the main qualitative conclusions reached in our study because (1) we excluded years with largely uneven seasonal searching effort, and (2) the seasonality in DMR estimated primarily from hatching dates should still be of biological relevance for the hatching dates themselves.

In summary, we provide the first correlative study to demonstrate the relationship between variation in nesting mortality and the inter-annual seasonal distribution of timing of breeding on the population level in birds with implication for climate change studies. Moreover, we show that the seasonal patterns in nesting mortality affect the seasonal distribution of the reproductive output of the whole population.

## Supporting Information

Figure S1 **Site-specific temporal variability in selected breeding parameters of the red-backed shrike.** Shown are fits of linear regressions with their 95% confidence intervals. Full line for site C. a) Mean hatching date (site C: b_year_±SE = −0.15±0.06, t = −2.43, F_1,29_ = 5.92, p = 0.021, r^2^ = 0.17), b) standardized differential between the mean hatching date and mean hatching date weighted by brood size (SD_HD_), c) mean cumulated total of degree days at hatching date with temperature threshold 0°C (mean CDD_HD_; site C: b_year_ = 3.74±1.68, t = 2.22, F_1,29_ = 4.95, p = 0.034, r^2^ = 0.15), and d) standardized seasonal linear trend in daily mortality rate (trendDMR). Site A: full circles, site B: full triangles, site C: empty circles and site D: empty triangles. 0 = 1 January.
(TIF)

Figure S2 **Site-specific effects of selected covariates on mean hatching date of the red-backed shrike.** Shown are fits of linear regressions, dot-dashed line for site B, full line for site C, long-dashed line for site D. a) Effect of TMAY; site B (bTMAY = −3.86±0.77, t = −5.03, F1,2 = 25.28, p = 0.037, r2 = 0.93), site C (bTMAY = −1.68±0.36, t = −4.69, F1,29 = 21.98, p<0.001, r2 = 0.43) and site D (bTMAY = −2.40±0.78, t = −3.07, F1,4 = 9.42, p = 0.037, r2 = 0.70). b) Effect of DMR; site C (bDMR = 164.17±89.04, t = 1.84, F1, 29 = 3.40, p = 0.076, r2 = 0.10). c) Effect of ln(density); site B (bln(density) = 12.40±3.08, t = 4.02, F1,2 = 16.18, p = 0.057, r2 = 0.89), site C (bln(density) = −5.53±1.64, t = −3.38, F1,29 = 11.40, p = 0.0021, r2 = 0.28). Site A: full circles, site B: full triangles, site C: empty circles and site D: empty triangles. 0 = 1 January.
(TIF)

Figure S3 **Site-specific effects of selected covariates on the seasonal distribution of reproductive output of the red-backed shrike.** Shown are fits of linear regressions with its 95% confidence intervals. See title of Fig. S1 and Fig. S2 for further explanations. a) The effect of mean CDD_HD_;_._ site B (b_CDDHD_ = −0.002±0.0001, t = −11.39, F_1,2_ = 129.8, p = 0.0076, r^2^ = 0.98). b) The effect of trendDMR. Only significant estimates of trendDMR, i.e. confidence intervals of the estimates exluded zero, were considered here and linear regression was fitted to data from all sites (n = 9) (b_trendDMR_ = −0.03±0.01, t = −2.84, F_1,7_ = 8.04, p = 0.025, r^2^ = 0.53). c) The effect of ln-density; site C (b_ln(density)_ = −0.06±0.03, t = −1.99, F_1,29_ = 3.96, p = 0.056, r^2^ = 0.12).
(TIF)

Table S1 Characteristics of the study sites in the Czech Republic.
(DOC)

Table S2 Summary of the breeding data in the red-backed shrike.
(DOC)

Table S3 Results of multiple linear regression models on mean hatching date at site C.
(DOC)

Results S1 Site-specific analyses.
(DOC)

Results S2 Associations between variables.
(DOC)
