# Supplementary figures and images for: Impact of Nesting Mortality on Avian Breeding Phenology: A Case Study on the Red-Backed Shrike (Lanius collurio)
Source: PLoS One. 2012 Aug 28;7(8):e43944. doi: 10.1371/journal.pone.0043944 (PMC3429440; doi:10.1371/journal.pone.0043944)

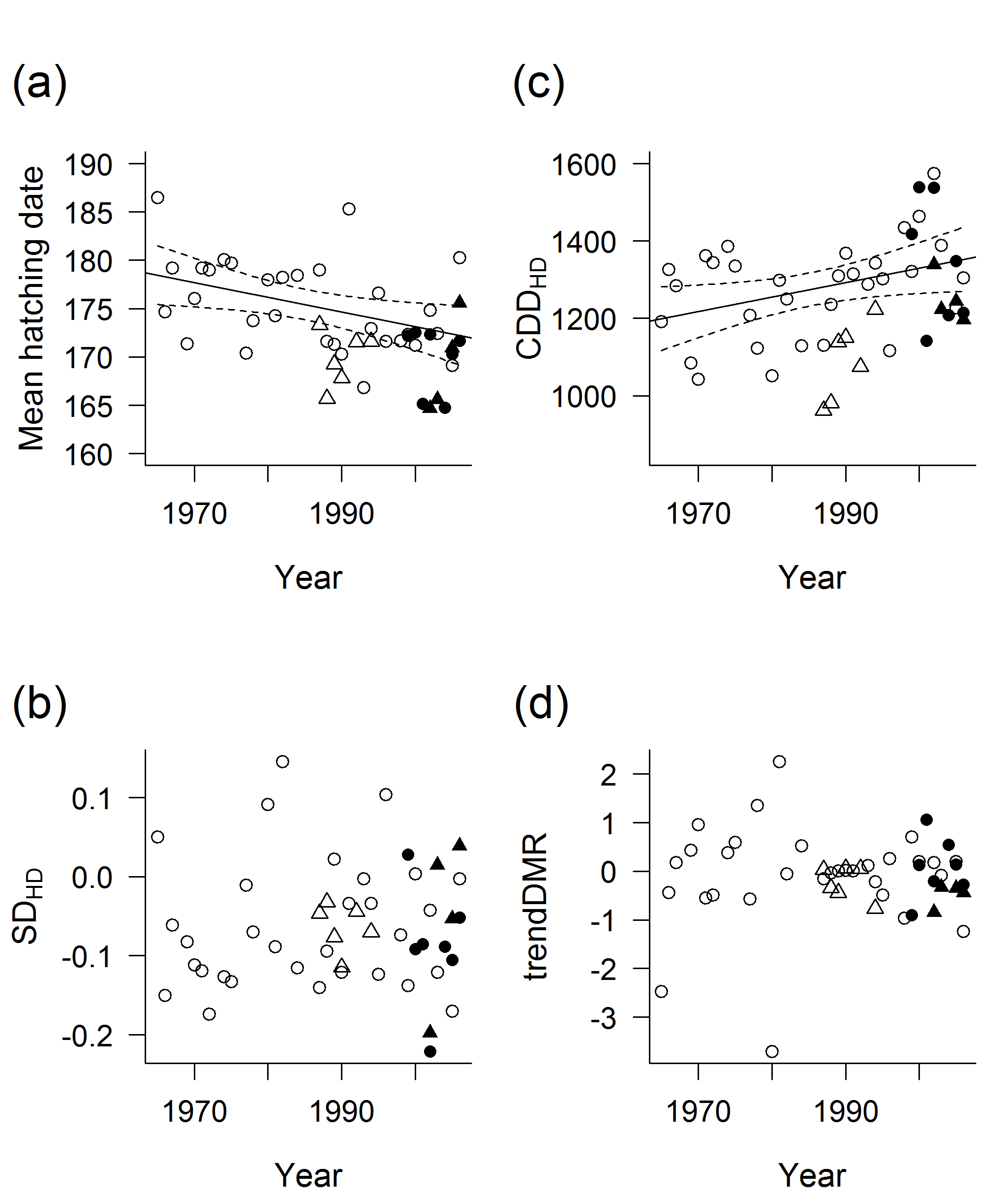

Supplement: Figure S1 — Site-specific temporal variability in selected breeding parameters of the red-backed shrike. Shown are fits of linear regressions with their 95% confidence intervals. Full line for site C. a) Mean hatching date (site C: byear±SE = −0.15±0.06, t = −2.43, F1,29 = 5.92, p = 0.021, r2 = 0.17), b) standardized differential between the mean hatching date and mean hatching date weighted by brood size (SDHD), c) mean cumulated total of degree days at hatching date with temperature threshold 0°C (mean CDDHD; site C: byear = 3.74±1.68, t = 2.22, F1,29 = 4.95, p = 0.034, r2 = 0.15), and d) standardized seasonal linear trend in daily mortality rate (trendDMR). Site A: full circles, site B: full triangles, site C: empty circles and site D: empty triangles. 0 = 1 January. (TIF) [file pone.0043944.s001.tif]

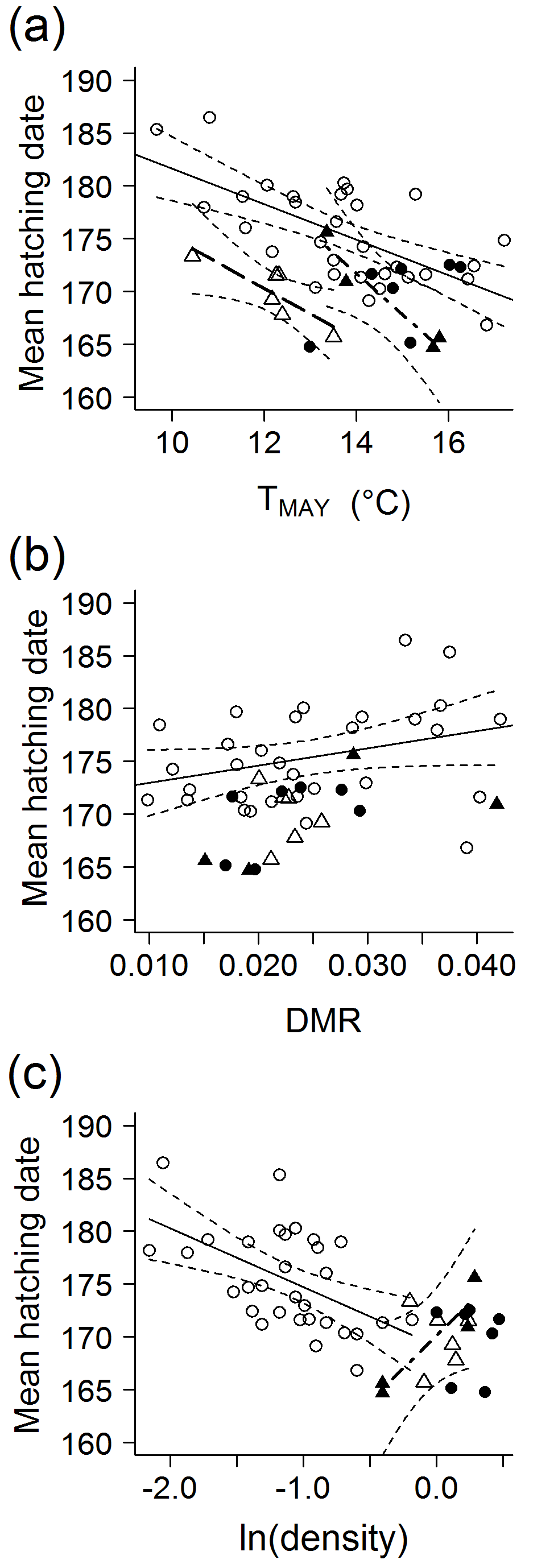

Supplement: Figure S2 — Site-specific effects of selected covariates on mean hatching date of the red-backed shrike. Shown are fits of linear regressions, dot-dashed line for site B, full line for site C, long-dashed line for site D. a) Effect of TMAY; site B (bTMAY = −3.86±0.77, t = −5.03, F1,2 = 25.28, p = 0.037, r2 = 0.93), site C (bTMAY = −1.68±0.36, t = −4.69, F1,29 = 21.98, p<0.001, r2 = 0.43) and site D (bTMAY = −2.40±0.78, t = −3.07, F1,4 = 9.42, p = 0.037, r2 = 0.70). b) Effect of DMR; site C (bDMR = 164.17±89.04, t = 1.84, F1, 29 = 3.40, p = 0.076, r2 = 0.10). c) Effect of ln(density); site B (bln(density) = 12.40±3.08, t = 4.02, F1,2 = 16.18, p = 0.057, r2 = 0.89), site C (bln(density) = −5.53±1.64, t = −3.38, F1,29 = 11.40, p = 0.0021, r2 = 0.28). Site A: full circles, site B: full triangles, site C: empty circles and site D: empty triangles. 0 = 1 January. (TIF) [file pone.0043944.s002.tif]

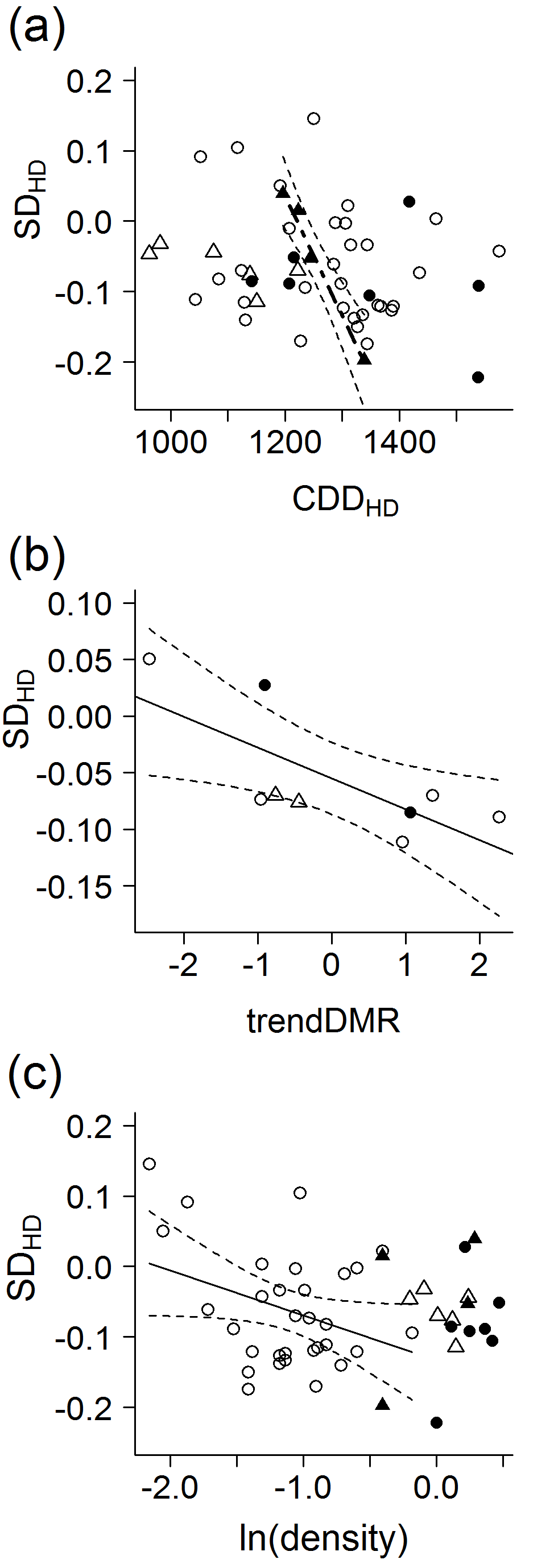

Supplement: Figure S3 — Site-specific effects of selected covariates on the seasonal distribution of reproductive output of the red-backed shrike. Shown are fits of linear regressions with its 95% confidence intervals. See title of Fig. S1 and Fig. S2 for further explanations. a) The effect of mean CDDHD;. site B (bCDDHD = −0.002±0.0001, t = −11.39, F1,2 = 129.8, p = 0.0076, r2 = 0.98). b) The effect of trendDMR. Only significant estimates of trendDMR, i.e. confidence intervals of the estimates exluded zero, were considered here and linear regression was fitted to data from all sites (n = 9) (btrendDMR = −0.03±0.01, t = −2.84, F1,7 = 8.04, p = 0.025, r2 = 0.53). c) The effect of ln-density; site C (bln(density) = −0.06±0.03, t = −1.99, F1,29 = 3.96, p = 0.056, r2 = 0.12). (TIF) [file pone.0043944.s003.tif]
