# Supplementary material for: Impact of Nesting Mortality on Avian Breeding Phenology: A Case Study on the Red-Backed Shrike (Lanius collurio)
Source: PLoS One. 2012 Aug 28;7(8):e43944. doi: 10.1371/journal.pone.0043944 (PMC3429440; doi:10.1371/journal.pone.0043944)
Supplement: Table S1 — Characteristics of the study sites in the Czech Republic. (DOC) [file pone.0043944.s004.doc]

Table S1. **Characteristics of the study sites in the Czech Republic.**

| Site | Location | Altitude (m) | Area (km^2^) |
| --- | --- | --- | --- |
| A | 49°59'N, 14°19'E | 300–350 | 25 |
| B | 49°51'N, 14°22'E | 300–400 | 15 |
| C | 50°29'N, 15°47'E | 400–500 | 52–78* |
| D | 49°22'N, 17°58'E | 350–500 | 100 |

*78 km^2^ during 1959 – 1996, 52 km^2^ during 1997 – 2006
