# Supplementary material for: Impact of Nesting Mortality on Avian Breeding Phenology: A Case Study on the Red-Backed Shrike (Lanius collurio)
Source: PLoS One. 2012 Aug 28;7(8):e43944. doi: 10.1371/journal.pone.0043944 (PMC3429440; doi:10.1371/journal.pone.0043944)
Supplement: Table S2 — Summary of the breeding data in the red-backed shrike. (DOC) [file pone.0043944.s005.doc]

Table S2. **Summary of the breeding data in the red-backed shrike.**

|  |  | Ringing data | | | Nest monitoring data | |
| --- | --- | --- | --- | --- | --- | --- |
| Site | Years* | Mean No. ringed broods | Mean HD** | Mean brood size** | Mean No. monitored nests | Range in annual DMR |
| A | 7 | 32.9 (25–40) | 170 (165 ­– 173) | 4.4 (3.8 – 4.9) | 40 (28–53) | 0.017 – 0.029 |
| B | 4 | 14.8 (10­–20) | 169 (165 – 176) | 4.1 (3.8 – 4.7) | 20.3 (9–31) | 0.015 – 0.042 |
| C | 31 | 26.1 (9–65) | 175 (167 – 187) | 4.1 (3.1 – 4.9) | 24.0 (9–63) | 0.010 – 0.042 |
| D | 6 | 105 (82–127) | 170 (166 – 173) | 4.6 (4.3 – 4.8) | 98.5 (72–125) | 0.020 – 0.026 |

* Site A: 1999–2002, 2004­­–2006

Site B: 2002–2003, 2005–2006

Site C: 1965–1967, 1969–1972, 1974–1975,1977–1978,1980­–1982,1984,1987–1991,1993–1996,1998–2000,2002–2003,2005–2006

Site D: 1987–1990,1992,1994

**Calculated from annual mean values.

See Fig. S1 for temporal variation in annual mean hatching date of survived first and replacement clutches (HD). Brood size refers to the ringing date. Seasonal mean daily mortality rates (DMR) was estimated from a subset of monitored nests. Note that in some cases the number of ringed broods was higher than the number of monitored nests because a portion of later found broods was ringed on the first visit. Such cases were included in the calculation of the breeding phenology only. Day 0 = 1 January. Shown in parentheses are the ranges of values per year.
