# Supplementary material for: Impact of Nesting Mortality on Avian Breeding Phenology: A Case Study on the Red-Backed Shrike (Lanius collurio)
Source: PLoS One. 2012 Aug 28;7(8):e43944. doi: 10.1371/journal.pone.0043944 (PMC3429440; doi:10.1371/journal.pone.0043944)
Supplement: Table S3 — Results of multiple linear regression models on mean hatching date at site C. (DOC) [file pone.0043944.s006.doc]

Table S3. **Results of multiple linear regression models on mean hatching date at site C.**

| explanatory variable | slope | SE | t | p |
| --- | --- | --- | --- | --- |
| *Multiple linear regression a** |  |  |  |  |
| **T_MAY_** | **-1.51** | **0.40** | **-3.77** | **< 0.001** |
| Year | -0.05 | 0.06 | -0.95 | 0.35 |
|  | | | | |
| *Multiple linear regression b*** |  |  |  |  |
| **DMR** | **180.81** | **80.59** | **2.24** | **0.033** |
| **Year** | **-0.16** | **0.06** | **-2.76** | **0.010** |

* F_2,28_ = 11.41, p < 0.001, R^2^ = 0.45

** F_2,28_ = 5.89, p = 0.0073, R^2^ = 0.30

Formal statistical significance at α = 0.05 is highlighted in bold.
