# Supplementary material for: Impact of Nesting Mortality on Avian Breeding Phenology: A Case Study on the Red-Backed Shrike (Lanius collurio)
Source: PLoS One. 2012 Aug 28;7(8):e43944. doi: 10.1371/journal.pone.0043944 (PMC3429440; doi:10.1371/journal.pone.0043944)
Supplement: Results S1 — Site-specific analyses. (DOC) [file pone.0043944.s007.doc]

Results S1**. Site-specific analyses**

**Trends in breeding parameters at individual sites**

At site C, the mean hatching date, advanced significantly over the years (Fig. S1a). Also, there was a linear trend to higher T_MAY_ (b_year_ = 0.06 ± 0.02, t = 3.05, p = 0.004) and faster spring progress at the time of red-backed shrike hatching time (mean CDD_HD_; Fig. S1c). There was no trend in either the distribution of the reproductive output relative to hatching dates (SD_HD_; Fig. S1b), nor in the seasonality in nesting mortality (trendDMR; Fig. S1d) (all p > 0.1). At site A, B and D, the long term trends were not evaluated because of short time series (Table 1, Fig. S1).

**Analysis of mean hatching date at individual sites**

Mean hatching date was significantly negatively related to T_MAY_ at site B, C and D but not at site A (p = 0.14; Fig. S2a). Only at site C delayed mean hatching date with higher DMR but the relationship was rather weak (Fig. S2b). No relationship was detected at other sites (all p > 0.2, Fig. S2b). The trend towards earlier mean hatching date was no longer significant at site C after accounting for the effect of T_MAY_ (Table S3). Mean hatching date, however, still advanced at site C even after the effect of DMR was accounted for (Table S3).

**Seasonality in reproductive output at individual sites**

Only at site B was the distribution of reproductive output skewed to higher brood sizes earlier in the season when the spring progressed faster at hatching time (Fig. S3a), but very small sample size prevented strong conclusions to be reached. The effect was not detectable at other sites (all p > 0.1). The distribution of reproductive output at site C was skewed to higher brood sizes hatched earlier in the season when the nesting mortality increased during the season (positive values of trendDMR, Fig. S3b) and breeding density was higher (Fig. S3c). The pattern was not detectable at other sites (all p > 0.3, Fig. S3a). Note that only significant estimates of trendDMR were included in the analysis.
