# Supplementary material for: Impact of Nesting Mortality on Avian Breeding Phenology: A Case Study on the Red-Backed Shrike (Lanius collurio)
Source: PLoS One. 2012 Aug 28;7(8):e43944. doi: 10.1371/journal.pone.0043944 (PMC3429440; doi:10.1371/journal.pone.0043944)
Supplement: Results S2 — Associations between variables. (DOC) [file pone.0043944.s008.doc]

Results S2**. Associations between variables**

DMR was not related to ln(density) at either site (all p > 0.1) nor across sites (LMM: b_ln(density)_ = -0.002 ± 0.002, t = -1.11, p = 0.27). DMR tended to be negatively related to T_MAY_ at site C (linear regression: b_TMAY_ = -0.001 ± 0.0009, t = -1.7, p = 0.096) but not at other sites (all p > 0.1). Similarly, the association was marginally not significant across sites (LMM: b_TMAY_ = -0.001 ± 0.0006, t = -1.7, p = 0.095). Neither ln(density) was related to T_MAY_ (LMM: b_TMAY_ = 0.04 ± 0.04, t = 1.03, p = 0.31).
